# Hair Growth Promoting and Anticancer Effects of p21-activated kinase 1 (PAK1) Inhibitors Isolated from Different Parts of *Alpinia zerumbet*

**DOI:** 10.3390/molecules22010132

**Published:** 2017-01-14

**Authors:** Nozomi Taira, Binh Cao Quan Nguyen, Shinkichi Tawata

**Affiliations:** 1 Department of Bioscience and Biotechnology, The United Graduate School of Agricultural Sciences, Kagoshima University, Korimoto 1-21-24, Kagoshima 890-8580, Japan; taira5935@gmail.com (N.T.); ncqbinh@gmail.com (B.C.Q.N.); 2 PAK Research Center, University of the Ryukyus, Okinawa 903-0213, Japan

**Keywords:** *Alpiniazerumbet*, PAK1 inhibitors, hair growth, cancer, kaempferol-3-*O*-β-d-glucuronide

## Abstract

PAK1 (p21-activated kinase 1) is an emerging target for the treatment of hair loss (alopecia) and cancer; therefore, the search for PAK1 blockers to treat these PAK1-dependent disorders has received much attention. In this study, we evaluated the anti-alopecia and anticancer effects of PAK1 inhibitors isolated from *Alpinia zerumbet* (alpinia) in cell culture. The bioactive compounds isolated from alpinia were found to markedly promote hair cell growth. Kaempferol-3-*O*-β-d-glucuronide (KOG) and labdadiene, two of the isolated compounds, increased the proliferation of human follicle dermal papilla cells by approximately 117%–180% and 132%–226%, respectively, at 10–100 μM. MTD (2,5-bis(1*E*,3*E*,5*E*)-6-methoxyhexa-1,3,5-trien-1-yl)-2,5-dihydrofuran) and TMOQ ((*E*)-2,2,3,3-tetramethyl-8-methylene-7-(oct-6-en-1-yl)octahydro-1*H*-quinolizine) showed growth-promoting activity around 164% and 139% at 10 μM, respectively. The hair cell proliferation induced by these compounds was significantly higher than that of minoxidil, a commercially available treatment for hair loss. Furthermore, the isolated compounds from alpinia exhibited anticancer activity against A549 lung cancer cells with IC_50_ in the range of 67–99 μM. Regarding the mechanism underlying their action, we hypothesized that the anti-alopecia and anticancer activities of these compounds could be attributed to the inhibition of the oncogenic/aging kinase PAK1.

## 1. Introduction

Hair loss (alopecia) is an ever-increasing trend, affecting the physical and mental health of both women and men [1,2]. Alopecia is not considered a disease; it is defined as the result of genetic disorders, nutritional and hormonal imbalance, aging, or stress [3,4]. Treatment for hair loss can be costly, ranging from simply wearing a wig, to more invasive medical treatments, such as human hair transplantation [2,5]. Drug treatment provides only a temporary solution, as its discontinuation causes hair loss to resume [5]. Although much effort has been devoted to developing new agents to treat hair loss over the past few years, so far only two drugs, finasteride and minoxidil, have been approved by the FDA [6]. Unfortunately, these two drugs have limited efficacy and exhibit undesirable side effects, such as pruritis, scaling, local irritation, dermatitis, and sexual dysfunction [4,7,8]. The other side of the medical spectrum is cancer, which takes the lives of millions of people per year worldwide, and is the second leading cause of death after cardiovascular disease [9]. No currently available anticancer drugs can eradicate cancer cells without harming normal tissue [10]. Herbal compounds have received much attention for the treatment of both hair loss and cancer because many of them, such as propolis, exert their therapeutic effect without side effects [11,12]. Therefore, searching for naturally-occurring new compounds to prevent hair loss and cancer is demanded and will offer social and economic benefits. 

PAK1 (p21-activated kinase 1) has been known to play a key role in many diseases and disorders including various cancers, neurofibromatosis (NF), type 2 diabetes mellitus, hypertension, pathologic shortened lifespan, and neurodegenerative diseases [13]. More than 70% of all human cancers, including breast and prostate cancers, RAS-induced pancreatic and colon cancers, and neurofibromatosis-associated tumors, are dependent on PAK1 for their growth and development [14]. Recently, the melanogenic role of PAK1 has been revealed by silencing the PAK1 gene in melanoma cells, explaining why a series of PAK1 blockers have been found to be useful for brightening skin [15]. Interestingly, some herbal PAK1 blockers such as curcumin, apigenin, and artepillin C from propolis, as well as 5,6-dehydrokawain (DK) and dihydro-5,6-dehydrokawain (DDK) from *Alpinia zerumbet*, were shown to promote hair cell growth [16,17,18], suggesting the possibility that PAK1 could suppress the growth of hair cells.

**Figure 1:**
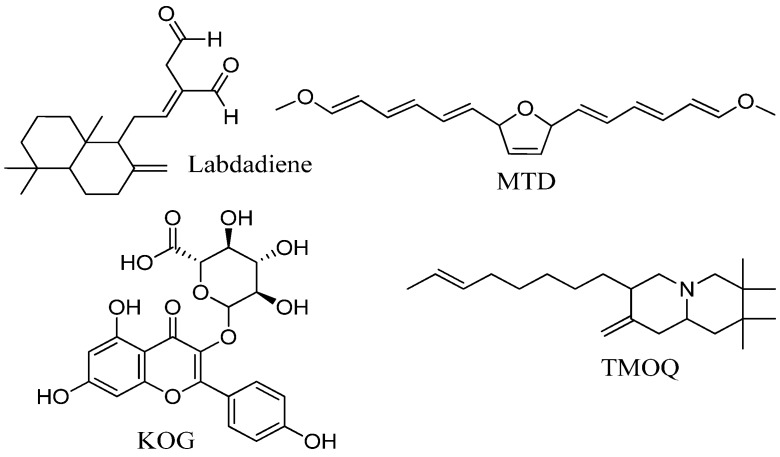
Chemical structures of isolated compounds in this study. Labdadiene: 8(17),12-Labdadiene-15,16-dial; MTD: 2,5-bis (1*E*,3*E*,5*E*)-6-methoxyhexa1,3,5-trien-1-yl)-2,5-dihydrofuran; KOG: kaempferol-3-*O*-β-d-glucuronide; TMOQ: (*E*)-2,2,3,3-tetramethyl-8-methylene-7-(oct-6-en-1-yl)octahydro-1*H*-quinolizine.

*Alpinia zerumbet* (family: Zingiberaceae) (alpinia), a perennial ginger that grows widely in the subtropical and tropical regions, has been used as a traditional medicine for its anti-inflammatory, bacteriostatic, and fungistatic properties [19]. Alpinia leaves have been used to prepare Okinawan traditional *mu-chi* food, which is used to prevent the common cold [20]. The essential oil from its leaves possesses relaxant and anti-spasmodic actions in the rat ileum [21]. In our recently reported studies, alpinia has been identified as a source of bioactive compounds with herbicidal, insecticidal, and fungicidal properties [22,23], and inhibition of HIV-1 integrase and neuraminidase [24], advanced glycation end products, and enzymes related to skin diseases [25,26]. Recently, several compounds of alpinia, such as DK and DDK, have been found to directly inhibit the oncogenic/aging kinase PAK1 and promote hair cell growth [18,27]. Since PAK1 is associated with both cancer and hair loss, and alpinia is a useful source of PAK1 inhibitors, we isolated and evaluated the effects of PAK1-blocking bioactive compounds from alpinia against alopecia and cancer (Figure 1) in the present study. 

## 2. Results

### 2.1. Effect of Extract and Isolated Compounds on Hair Cell Growth

**Figure 2:**
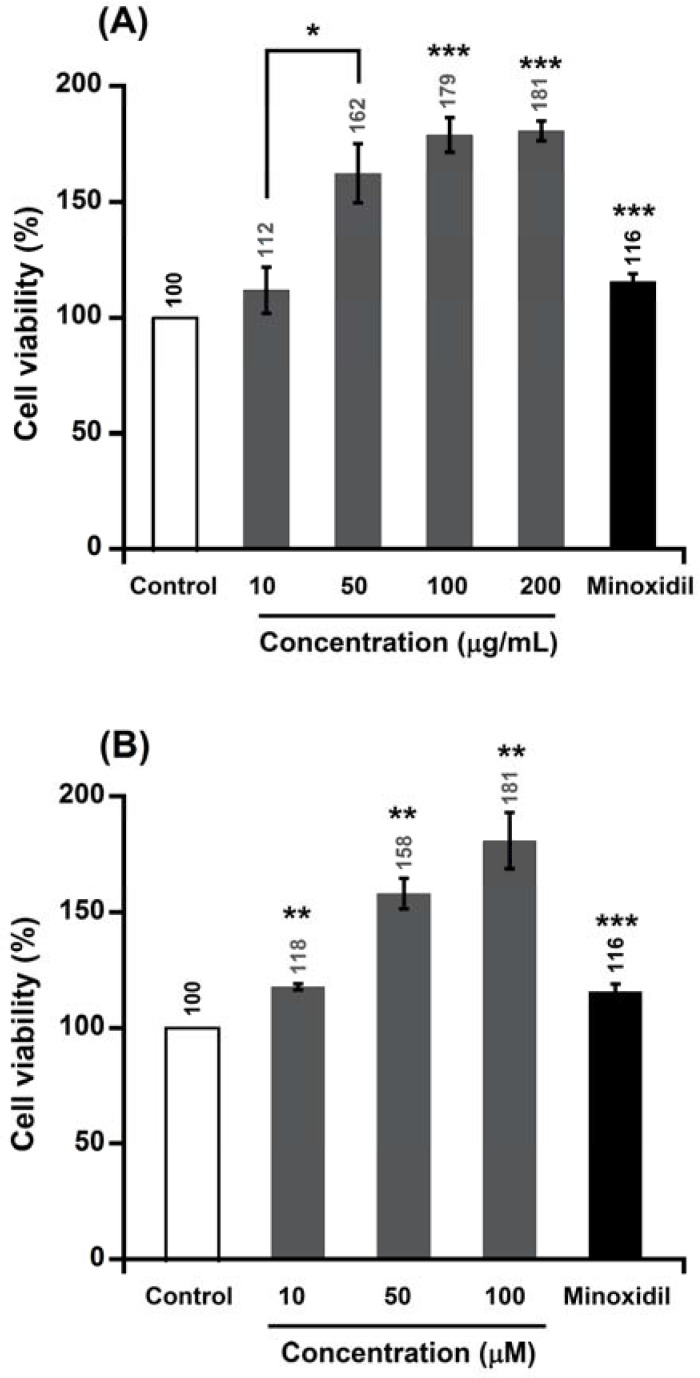
Effect of ALEB extract and kaempferol-3-*O*-β-d-glucuronide on the proliferation of human follicle dermal papilla cell (HFDPC). (**A**) ALEB, and (**B**) kaempferol-3-*O*-β-d-glucuronide (KOG). Results are the mean ± SE of six replications for each treatment. Minoxidil (10 μM) was used as a positive control. Asterisks indicate significant difference between the treatment and control. * 0.01 ≤ *p* ≤ 0.05; ** *p* < 0.01; *** *p* < 0.001.

**Figure 3:**
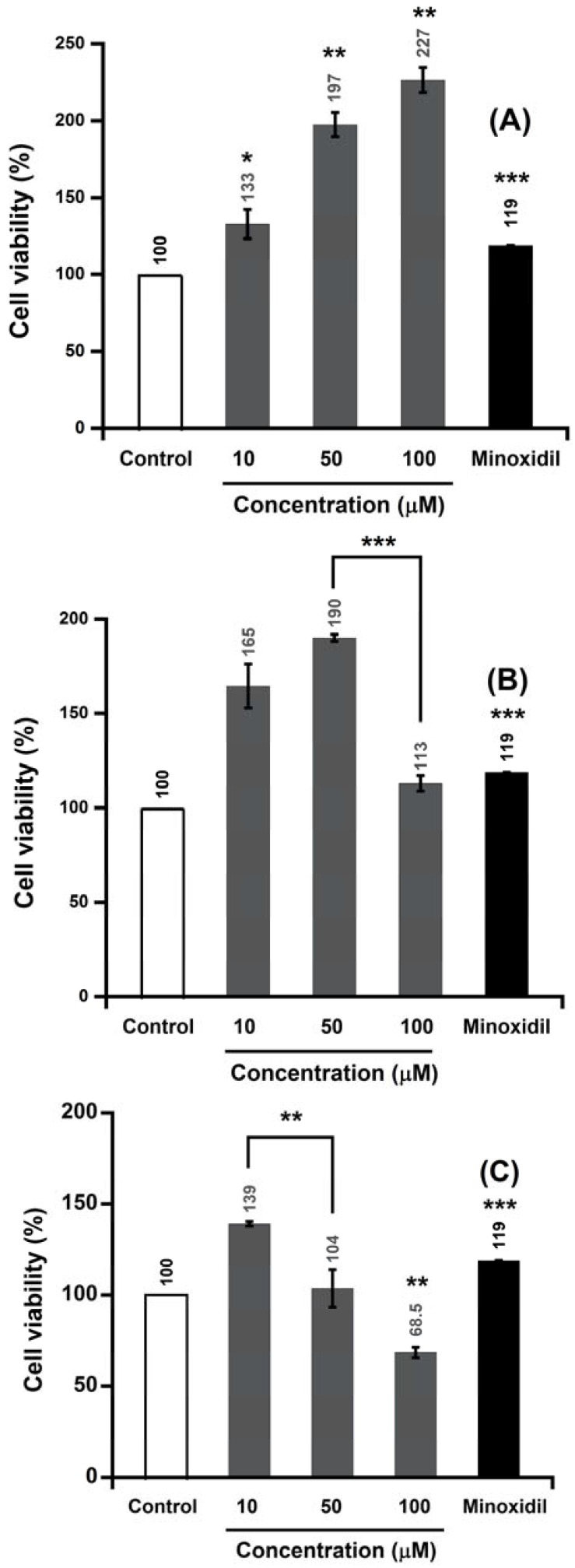
Effect of labdadiene, MTD, and TMOQ on the proliferation of human follicle dermal papilla cells (HFDPC). (**A**) Labdadiene, (**B**) MTD, and (**C**) TMOQ. Results are the mean ± SE of six replications for each treatment. Minoxidil (10 μM) was used as a positive control. Asterisks indicate significant difference between treatment and control. * 0.01 ≤ *p* ≤ 0.05; ** *p* < 0.01; *** *p* < 0.001.

The potential of alpinia extract and isolated compounds to promote the growth of human follicle dermal papilla cells (HFDPC) was evaluated by the thiazolyl blue tetrazolium bromide (MTT) assay. As shown in Figure 2, the ALEB (ethyl acetate and butanol extract) of alpinia leaves increased the proliferation of hair cells about 111%–180% at 10–200 μg/mL. Alpinia leaves are a source of a wide range of bioactive constituents. Previous studies have reported the presence of flavonoid compounds in alpinia leaves [28]. Some flavonoids, such as baicalin, apigenin, and quercetin, promoted hair growth in a mouse model or in cell culture [16,29,30]. Therefore, we hypothesized that hair cell growth-promoting activity of ALEB extract may be attributed to the flavonoid compounds. After purification and measurement of proliferative activity with respect to hair follicle cells, kaempferol-3-*O*-β-d-glucuronide (KOG), a flavonoid compound, was identified as one of the active compounds in the ALEB extract responsible for increasing cell proliferation. KOG increased the proliferation of HFDPC by approximately 117%, 158%, and 180% at 10, 50, and 100 μM, respectively (Figure 2). In contrast, labdadiene increased the proliferation of hair cells by about 132%, 197%, and 226% at 10, 50, and 100 μM, respectively. Interestingly, at 10 μM concentration, both compounds 2,5-bis (1*E*,3*E*,5*E*)-6-methoxyhexa1,3,5-trien-1-yl)-2,5-dihydrofuran (MTD) and (*E*)-2,2,3,3-tetramethyl-8-methylene-7-(oct-6-en-1-yl)octahydro-1*H*-quinolizine (TMOQ) increased HFDPC proliferation around 164% and 139%, respectively (Figure 3). All of these compounds had better proliferative activity compared with minoxidil at 10 μM. 

### 2.2. Anticancer Activity

The anticancer activity of isolated compounds was evaluated by MTT assay. As shown in Table 1, among tested compounds, labdadiene showed the strongest inhibitory activity against the PAK1-dependent growth of A549 lung cancer cells with an IC_50_ of 67 μM. The IC_50_ of the remaining compounds are between 81 and 99 μM. However, none of them is more potent than curcumin (IC_50_ = 30 μM). 

### 2.3. Direct Inhibition of PAK1 in Vitro

To further investigate the mechanism underlying their anticancer and anti-alopecia activities, their direct inhibition of PAK1 was evaluated in vitro. As shown in Table 2, KOG inhibited PAK1 with an IC_50_ of 39 μM, while labdadiene, MTD, and TMOQ resulted in IC_50_ values of 52, 59, and 49 μM, respectively. However, none of them is more potent than curcumin (IC_50_ = 13 μM).

## 3. Discussion

Hair growth is a cyclic process with an interplay between three continuous stages: anagen, catagen, and telogen [31]. In this cycle, the dermal papilla, located at the bottom of the hair follicle, is the most important element, and plays a major role in the formation of new hair follicles and the regulation of hair growth [1,32]. The most feasible and useful method for evaluating hair growth promotion is by determining the dermal papilla growth rate as influenced by various compounds [1]. As reported previously, several substances have been found to facilitate hair growth, all via different pathways. Finasteride stimulates hair growth by inhibiting steroid-5α-reductase, which catalyzes the conversion of testosterone into dihydrotestosterone [33]. Minoxidil extends anagen by activating beta-catenin signaling and the opening of ATP-sensitive potassium channels in dermal papilla cells [34,35]. It was also found to stimulate hair growth via the up-regulation of vascular endothelial growth factor (VEGF) [36]. However, there were a few cell culture-based studies suggesting that herbal PAK1-blockers such as curcumin, apigenin, and artepillin C from propolis to promote the growth of hair cells [16,17]. Thus, there is the possibility that PAK1 could normally suppress the growth of hair cells. Taken together, in the present study, KOG, labdadiene, and MTD demonstrated anti-alopecia activity, which could be the result of blocking of PAK1 by these compounds. However, although TMOQ inhibited PAK1 with an IC_50_ around 50 μM, TMOQ at this concentration or higher did not significantly affect the hair cell proliferation. Thus, it is unlikely that the hair growth-promoting activity of TMOQ at 10 μM is associated with PAK1 inhibition. 

Despite rapid growth of the field of drug discovery due to the use of synthetic and combinational approaches, naturally-occurring compounds still contribute valuable raw materials, especially in the area of cancer prevention and treatment. For example, 60% of all the approved chemotherapeutic cancer agents were derived from naturally-occurring compounds [37,38]. The isolated compounds from alpinia exhibited anticancer activity attributed to inhibition of PAK1, which is essential for the growth of A549 cells. The molecular mechanisms and the structure-activity relationship could be studied in perspective research to clarify how these compounds isolated from alpinia inhibit PAK1. However, to be useful for further clinical applications, chemical modification of these compounds is needed to produce far more potent derivatives with improved anticancer activity.

## 4. Materials and Methods 

### 4.1. Chemicals and Instrumental Analysis 

Fetal calf serum (FCS), cyproterone (Cyp), insulin transferring triiodothyronine (ITT), and bovine pituitary extract (BPE) were purchased from TOYOBO (Osaka, Japan). Dulbecco’s modified minimum essential medium (D-MEM) was purchased from Wako Pure Chemical Industries (Osaka, Japan). Fetal bovine serum (FBS) was obtained from HyClone (Victoria, Australia). Thiazolyl blue tetrazolium bromide (MTT) was obtained from Sigma-Aldrich (Saint Louis, MO, USA). ADP-Glo™ kinase assay kit was obtained from Promega (Madison, WI, USA). Unless otherwise mentioned, all reagents were of analytical grade and were obtained from Wako Pure Chemical Industries and Kanto Chemical Co. (Tokyo, Japan). ^1^H-NMR and ^13^C-NMR spectra were obtained on an ULTRASHIELD PLUS 400 MHz (Bruker Biospin, Rheinstetten, Germany). Chemical shifts were expressed in parts per million (*δ*) relative to tetramethylsilane (TMS).

### 4.2. Preparation of the Extracts and Isolation of Compounds

Fresh alpinia leaves (250 g) were extracted with boiling water (500 mL) for 15 min. The cooled extract was filtered and partitioned successively with hexane, dichloromethane, and chloroform. The extract was then fractioned continuously with ethyl acetate and butanol to give a crude extract (1.2 g) (ALEB). The ALEB extract was subjected to chromatography column on a Sephadex LH-20 with ethanol/acetone (19:1) to give two fractions. Fraction 1 was subjected to preparative thin-layer chromatography (PTLC) with butanol/acetic acid/water solvent (6:1:2). Compound kaempferol-3-*O*-β-d-glucuronide (KOG) was isolated by high-performance liquid chromatography (HPLC) in a pure state (Shimadzu, Kyoto, Japan). The mobile phase was 0.1% acetic acid in distilled water (solvent A), and 0.1% acetic acid in methanol (solvent B). HPLC conditions were as follows: 0–27 min, 10% solvent B; 27–30 min, 90% solvent B. HREIMS: *m/z* 463.0 [M + H]^+^ (calcd for C_21_H_18_O_12_, 462.3). ^1^H-NMR (400 MHz, MeOD-*d4*) *δ* 3.32–3.78 (m, 1H, sugar), 5.38 (d, 1H, CH, *J* = 7.5 Hz), 6.22 (s, 1H, CH), 6.42 (s, 1H, CH), 6.89 (d, 2H, CH, *J* = 8.8 Hz,), 8.10 (d, 2H, CH, *J* = 8.8 Hz). ^13^C-NMR *δ* 71.9, 74.4, 76.1, 76.3, 94.1, 99.2, 101.5, 104.3, 115.5, 121.1, 131.4, 133.5, 156.8, 160.5, 161.6, 164.7, 169.9, 177.7. 

Labdadiene was isolated from the hexane extract of the rhizomes using the method described previously [25]. Briefly, the dried rhizomes were extracted with hexane for 48 h. The crude extract was further separated using silica gel column chromatography using hexane/acetone (9:1). The aliquots were separated using a Diaion HP-20 resin column (Mitsubishi Chemical Co., Tokyo, Japan) with methanol gradient 50%–100%, and further purified by HPLC (Shimadzu, Kyoto, Japan) to acquire labdadiene. EIMS *m/z* (Rel. int); 302 (20), 137 (100), 123 (50), 109 (35), 95 (73), 81 (70), 69 (55), 55 (48), 41 (50). ^1^H-NMR (400 MHz, CDCl_3_) *δ* 0.73 (s, 3H, CH_3_), 0.82 (s, 3H, CH_3_), 0.89 (s, 3H, CH_3_), 1.04–2.52 (m, 14H, CH_2_, CH), 3.49 (s, 2H, CH_2_), 4.37 (s, 1H, CH_2_), 4.86 (s, 1H, CH_2_), 6.76 (t, 1H, CH), 9.40 (s, 1H, CHO), 9.63 (t, 1H, CHO). ^13^C-NMR *δ* 14.4, 19.4, 21.7, 24.2, 33.6, 37.9, 39.3, 39.4, 39.6, 42.0, 55.4, 56.7, 108.0, 134.9, 148.0, 160.1, 193.7, 197.5. 

2,5-bis(1*E*,3*E*,5*E*)-6-methoxyhexa-1,3,5-trien-1-yl)-2,5-dihydrofuran (MTD) was isolated by another group in our laboratory [39]. Dried rhizomes (1000 g) were extracted with 1.5 L ethanol for two days at room temperature. The suspension was filtered, and the filtrate was evaporated under reduced pressure. The crude extract was dissolved in 300 mL distilled water and the fat extracted with 300 mL hexane (defatted). The defatted aqueous extract was fractionated with 200 mL chloroform, and then 200 mL ethyl acetate. The ethyl acetate fraction was subjected to silica gel column chromatography with petroleum ether/chloroform (0%–100%) to afford three fractions. Fraction 2 was further purified by HPLC to give pure MTD. The isolated compounds were collected at 280 nm using a Synergi 4μM MAX-RP 80 Å column (150 mm × 4.60 mm, 4 micron; Phenomenex, Torrance, CA, USA). The mobile phases were water with 0.1% acetic acid (solvent A) and acetonitrile with 0.1% acetic acid (solvent B) at a flow rate of 1 mL/min. The gradient elution was performed as follows: 0–7 min, 40%–70% B; 7–20 min, 70%–100% B; 20–30 min, 100% B. Analytical data are in agreement with previously reported data [39]. 

(*E*)-2,2,3,3-tetramethyl-8-methylene-7-(oct-6-en-1-yl)octahydro-1*H*-quinolizine (TMOQ) was also prepared by other group in our laboratory [39]. The seeds of alpinia (100 g) were extracted with 500 mL of methanol for two days. The filtrate was evaporated and suspended in 500 mL distilled water. The suspension was partitioned with 500 mL hexane, and then 500 mL ethyl acetate. The ethyl acetate fraction was subjected to a silica gel column chromatography with methanol in dichloromethane (1%–50%) to give four fractions. Fraction 4 was further purified using the same column and conditions described above to give pure TMOQ. Analytical data are in agreement with previously reported data [39]. 

### 4.3. Assay for in Vitro Hair Cell Growth Promotion 

Human follicle dermal papilla cell (HFDPC) (TOYOBO, Tokyo, Japan) proliferation was assayed as described by Nguyen et al. [18]. Cells were cultured in growth medium containing 50 mL papilla cell growth medium (PCGM), 0.5 mL FCS, 0.5 mL BPE, 0.25 mL Cyp, and 0.25 mL ITT (51.5 mL total volume). Cell viability was evaluated using the thiazolyl blue tetrazolium bromide (MTT) assay. Cells were collected and diluted in medium containing D-MEM and 10% FBS at a cell density of 1 × 10^4^ cells/mL. Then, cell suspension (200 μL) was transferred into a collagen-coated 96-well plate at a density of 2000 cells/well. After incubation for three days, 200 μL of the isolated compounds in DMEM was added. After four days of incubation, 100 μL of MTT solution in D-MEM (0.4 mg/mL) was added, and the mixture was incubated for 2 h. The untransformed MTT was removed, and 100 μL of 2-propanol was added to each well to dissolve the formazan crystals. The absorbance was read at 570 nm and 650 nm using a microplate reader. The cell viability was calculated from the readings, and represented as a percentage of the control value (cells treated with D-MEM only).

### 4.4. Anticancer Activity by MTT Assay 

The assay was performed as previously described [40]. Lung cancer cells (A549) were seeded into a 96-well plate at a density of 1 × 10^4^ cells/well, and cultured for 24 h before the compounds were added. The cells were incubated in a humidified atmosphere of 5% CO_2_ at 37 °C for 72 h. After treatment, 20 μL MTT solution (0.5 mg/mL) was added to each well, and the plates were incubated for 3 h. The medium was removed, and the formazan was dissolved in 200 μL of DMSO. After shaking the plate for 10 min, cell viability was assessed by measuring the absorbance at 490 nm using a microplate reader (Corona, Hitachinaka-ku, Ibaraki, Japan). DMSO was used as a blank. The corrected absorbance of sample was divided by the corrected absorbance of the control, and then multiplied by 100% to give the percentage cell viability.

### 4.5. In Vitro Assay for PAK1 Inhibition

PAK1 kinase activity was measured using the ADP-Glo™ kinase assay kit (Promega, Madison, WI, USA) according to manufacturer’s instructions. Briefly, human PAK1 (25 ng/reaction) was incubated with test compounds (5 μL) for 10 min. The kinase reaction was started by adding 2.5× adenosine triphosphate (ATP)/substrate mix (10 μL). The reaction was terminated by adding 25 μL ADP-Glo™ reagent, followed by an incubation time of 40 min. The kinase detection reagent (50 μL) was added to the reaction mixture, and after 30 min of incubation, the luminescence was recorded by MTP-880Lab microplate reader (Corona, Ibaraki, Japan) with an integration time of 0.5 s per well. The percentage inhibition was calculated relative to the control kinase activity without any inhibitor.

### 4.6. Data Analysis 

Data significance was assessed by one-way ANOVA analysis using Statistical Analysis System (SAS) software, version 9.1.3 (SAS Institute Inc., Cary, NC, USA). All calculations were conducted in Microsoft Excel 2003. IC_50_ values were determined graphically and represented 50% inhibition of the tested compound. *p* < 0.05 was interpreted as statistically significant. 

## 5. Conclusions

In the present study, we have described a few compounds derived from the different parts of *Alpinia zerumbet* and their outstanding anti-alopecia and anticancer effects in cell culture. These properties could be due to the inhibition of oncogenic/aging kinase PAK1 by the isolated compounds. In an attempt to prove this notion further, we are planning to test the effect of PAK1 gene silencing on the growth of hair cells. Nevertheless, our findings suggest that some of these alpinia-derived compounds have the potential to be leading compounds for the production of more potent therapeutics for alopecia and cancer. 

## Figures and Tables

**Table 1:** Anti-cancer activity of isolated compounds from *Alpinia zerumbet* against the A549 cell line.

Compound	IC_50_ (μM)
Labdadiene	67.1 ± 6.0 ^b^
MTD	98.9 ± 9.3 ^a^
TMOQ	90.8 ± 1.6 ^a^
KOG	81.4 ± 1.9 ^a,b^
Curcumin	30.3 ± 2.9 ^c^

Data have statistical significance at *p* ≤ 0.05. The results are the mean ± SE of six replications for each treatment. Various letters in the same column indicate statistically significant difference.

**Table 2:** In vitro PAK1 inhibitory activity of isolated compounds from *Alpinia zerumbet.*

Compound	IC_50_ (μM)
Labdadiene	52.1 ± 3.0 ^a,b^
MTD	58.6 ± 2.5 ^a^
TMOQ	49.3 ± 0.7 ^b,c^
KOG	39.3 ± 2.4 ^c^
Curcumin	12.9 ± 1.1 ^d^

Data have statistical significance at *p* ≤ 0.05. The results are the mean ± SE of six replications for each treatment. Various letters in the same column indicate statistically significant difference.
